# Genomic epidemiology of SARS- CoV-2 Omicron variants in the Republic of Korea

**DOI:** 10.1038/s41598-022-26803-w

**Published:** 2022-12-27

**Authors:** Dong-Wook Lee, Jeong-Min Kim, Ae Kyung Park, Da-Won Kim, Ji-Yun Kim, Noori Lim, Hyeokjin Lee, Il-Hwan Kim, Jeong-Ah Kim, Chae young Lee, Jung-Hoon Kwon, Eun-Jin Kim

**Affiliations:** 1 College of Veterinary Medicine, Kyungpook National University, Daegu, 41566 Republic of Korea; 2 Division of Emerging Infectious Diseases, Bureau of Infectious Disease Diagnosis Control, Korea Disease Control and Prevention Agency, Cheongju-Si, 28159 Republic of Korea

**Keywords:** Infectious diseases, Virology, SARS-CoV-2, Viral epidemiology

## Abstract

The outbreak of severe acute respiratory syndrome coronavirus 2 (SARS-CoV-2) has caused a global pandemic since 2019. Variants of concern (VOCs) declared by the World Health Organization require continuous monitoring because of their possible changes in transmissibility, virulence, and antigenicity. The Omicron variant, a VOC, has become the dominant variant worldwide since November 2021. In the Republic of Korea (South Korea), the number of confirmed cases increased rapidly after the detection of Omicron VOC on November 24, 2021. In this study, we estimated the underlying epidemiological processes of Omicron VOC in South Korea using time-scaled phylodynamic analysis. Three distinct phylogenetic subgroups (Kor-O1, Kor-O2, and Kor-O3) were detected in South Korea. The Kor-O1 subgroup circulated in the Daegu region, whereas Kor-O2 and Kor-O3 circulated in Incheon and Jeollanam-do, respectively. The viral population size and case number of the Kor-O1 subgroup increased more rapidly than those of the other subgroups, indicating the rapid spread of the virus. The results indicated the multiple introductions of Omicron sub-lineages into South Korea and their subsequent co-circulation. The evolution and transmission of SARS-CoV-2 should be continuously monitored, and control strategies need to be improved to control the multiple variants.

## Introduction

Severe acute respiratory syndrome coronavirus 2 (SARS-CoV-2) has spread globally; as of March 2, 2022, there have been 437,333,859 confirmed cases, including 5,960,972 deaths worldwide^1^. New variants of SARS-CoV-2 should be constantly observed because some contain mutations that increase their transmissibility, virulence, and ability to evade immune responses^2–4^. These variants have been classified as Variants of Concern (VOCs) by the World Health Organization^5^. These VOCs include B.1.1.7(Alpha), first identified in England^6^, B.1.351(Beta) in South Africa^7^, P.1(Gamma) in Brazil^8^, B.1.617.2(Delta) in India^9^, and B.1.1.529(Omicron) in South Africa and Botswana^10,11^. Global data show that the Alpha variant was the dominant variant until May 2021. Thereafter, the number of Delta variants increased and peaked from October 2021 to November 2021. After the emergence of Omicron in November 2021, the Omicron variant became the dominant global variant, and the weekly number of cases rapidly increased^12,13^. Omicron has many mutations compared with other variants. In particular, the mutations concentrated in the S protein enable it to evade neutralizing antibodies or vaccine-induced immunity^14–16^.

In the Republic of Korea (South Korea), the number of confirmed cases has continuously increased since the first influx of Omicron on November 24, 2021, surpassing 10,000 on January 26, 2022, and by mid-March 2022 more than 300,000 cases had been reported^17^. The increased transmissibility of Omicron VOC was suspected as a major factor in the exponential increase of cases in South Korea and worldwide; however, other factors, including vaccine efficacy and level of social distancing, may contribute to the viral spread. With an ongoing exponential increase in confirmed cases, simultaneous outbreaks occurring in all states of South Korea, and the continuous introduction of SARS-CoV-2 from abroad, tracing all the contacts of infected patients is impossible. Thus, epidemiological investigations based on contact tracking methods could not present significant epidemiological patterns of viruses. Consequently, molecular epidemiology based on phylogenetic analysis has become critical for understanding the outbreak of SARS-CoV-2. To investigate the recently increasing number of Omicron strains circulating in South Korea, we investigated the evolutionary and geographical relationships of Omicron in South Korea, based on the phylogenetic analysis of full-length viral genome sequences.

## Results

### Maximum-likelihood phylogenetic tree reveals three different virus groups transmitted in South Korea

**Figure 1:**
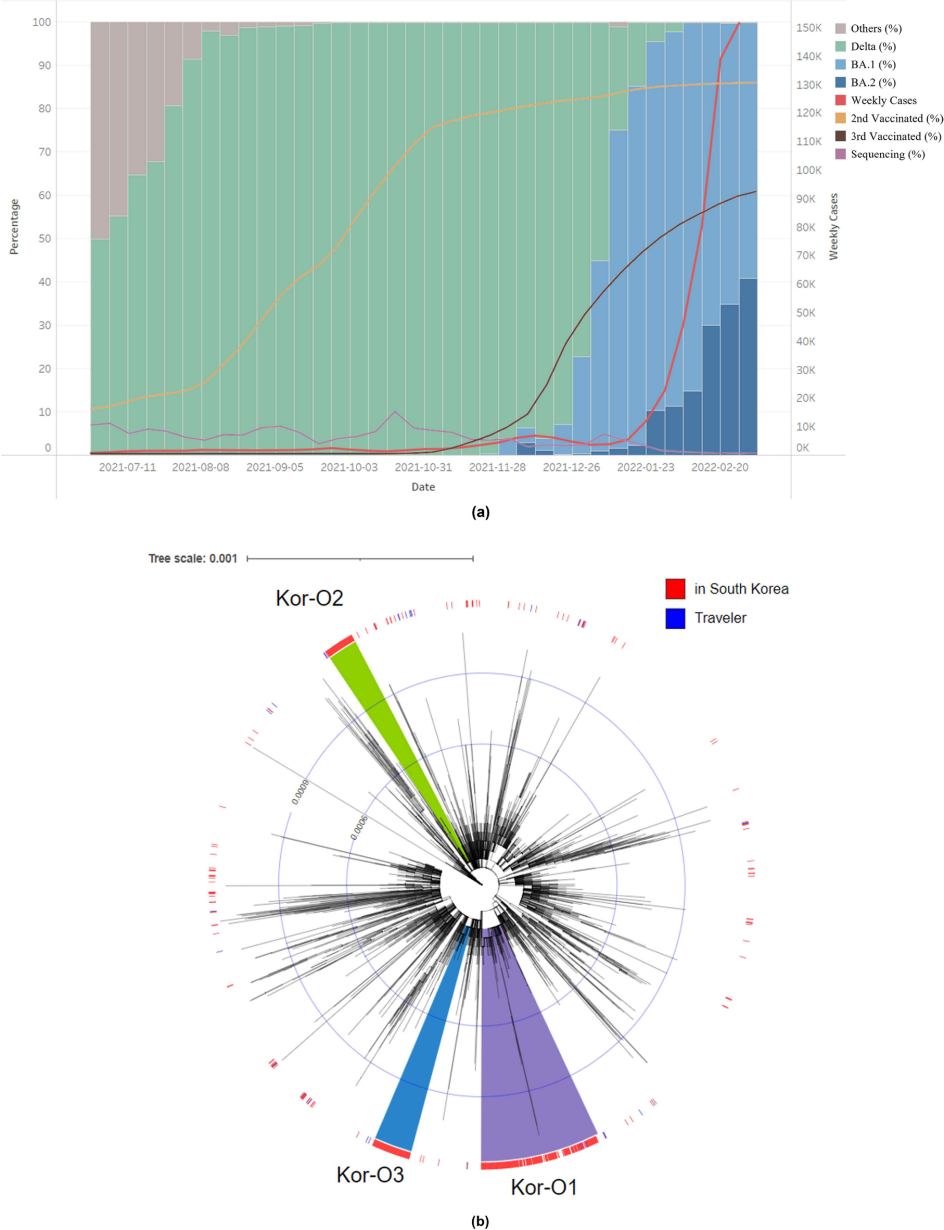
(**a**) Proportion of lineages and number of weekly cases of Korean sequences. Each column indicates the proportion of SARS-CoV-2 lineages of Korean sequences from July 7, 2021, to February 20, 2022. The red line indicates the number of weekly cases in South Korea from July 7, 2021, to February 20, 2022, and the yellow, brown, and purple lines indicate the 2nd vaccination rate, 3rd vaccination rate, and sequencing percentage. (**b**) Maximum-likelihood phylogenetic tree constructed by FastTree of 3330 foreign sequences and 582 Korean sequences. The SARS-CoV-2 viruses detected from travelers entering South Korea or their direct contacts were annotated by blue color strips, and viruses detected from South Korean residents with no travel history were annotated by red color strips. The Kor-O1 subgroup is highlighted in purple, Kor-O2 is highlighted in green, and Kor-O3 is highlighted in blue.

A total of 582 sequences of the Omicron variant were obtained from November 24, 2021, to January 11, 2022. The proportion of the Omicron variant rapidly increased during this period and replaced the previously dominant Delta variant in South Korea. Of the various sub-lineages, the BA.1 sub-lineage was dominant (Fig. 1a).

We constructed approximately maximum-likelihood phylogenetic trees of BA.1 sub-lineages and detected 128 distinct monophyletic subgroups sharing a common ancestral node with other Korean viruses, with a local support value of > 0.7. Most subgroups caused only single or short local outbreaks; however, three subgroups—Kor-O1 (n = 273), Kor-O2 (n = 68), and Kor-O3 (n = 86)—caused multiple local outbreaks in South Korea (Fig. 1b). In the Kor-O1 subgroup, two inbound travelers were detected, which included imported cases from Ivory Coast on December 30, 2021, and Congo on December 29, 2021; however, no inbound travelers were detected in the Kor-O2 or Kor-O3 subgroups (Table S1). The Kor-O1 subgroup also included 35 sequences detected outside South Korea, indicating the possible spread of viruses from South Korea to other countries.

The Kor-O1 and Kor-O3 subgroups belong to the BA.1.1 sub-lineage of Omicron, and the Kor-O2 subgroup belongs to BA.1. Korean Omicron sub-lineages have additional mutations from the original Omicron strain, hCoV-19/South Africa/NICD-N20868/2021, collected on November 11, 2021. All three subgroups have T5730C in ORF 1a and T462A in the ORF3a gene. The Kor-O1 subgroup mutated A603T and G604C in the N gene, Kor-O2 mutated G955A in the N gene, and The Kor-O3 subgroup mutated C2470T in the ORF 1a gene and C473T in the M gene (Fig. S1).

### Genomic epidemiology reveals that these three subgroups have different tendencies for geographic transmission

To investigate the time to most recent common ancestor (tMRCA), mutation rates, and transmission trends, we constructed a time-scaled phylogenetic tree. The tMRCA results showed that all three Korean subgroups were introduced into South Korea between mid-November and mid-December, and Kor-O2 was estimated to be the first subgroup. For the Kor-O1 and Kor-O3 subgroups, the difference between the first isolation date and the mean of tMRCA was 9 days, and for Kor-O2, it was 12 days. The mutation rates for all three subgroups were between 1.0 × 10^−3^ and 1.3 × 10^−3^ substitutions/site/year and did not show significant differences between the subgroups (Table 1).

**Table 1:** First detection date, mutation rates, and time to most recent common ancestor of each Korean Omicron subgroup.

Subgroup	Date of first detection in South Korea	tMRCA^1^ (95% HPD)^2^	Mutation rate^3^ (95% HPD)
Kor-O1	Dec. 21, 2021	Dec. 12, 2021 (Nov. 29, 2021–Dec. 19, 2021)	1.2227 × 10^−3^ (7.2495 × 10^−4^–1.9052 × 10^−3^)
Kor-O2	Nov. 29, 2021	Nov. 17, 2021 (Oct. 27, 2021–Nov. 28, 2021)	1.2952 × 10^−3^ (3.8827 × 10^−4^–2.6098 × 10^−3^)
Kor-O3	Dec. 06, 2021	Nov. 27, 2021 (Nov. 21, 2021–Dec. 3, 2021)	1.0314 × 10^−3^ (5.2968 × 10^−4^–1.6441 × 10^−3^)

^1^Time to most recent common ancestor.

^2^95% HPD, 95% height posterior density.

^3^Substitutions/site/year.

**Figure 2:**
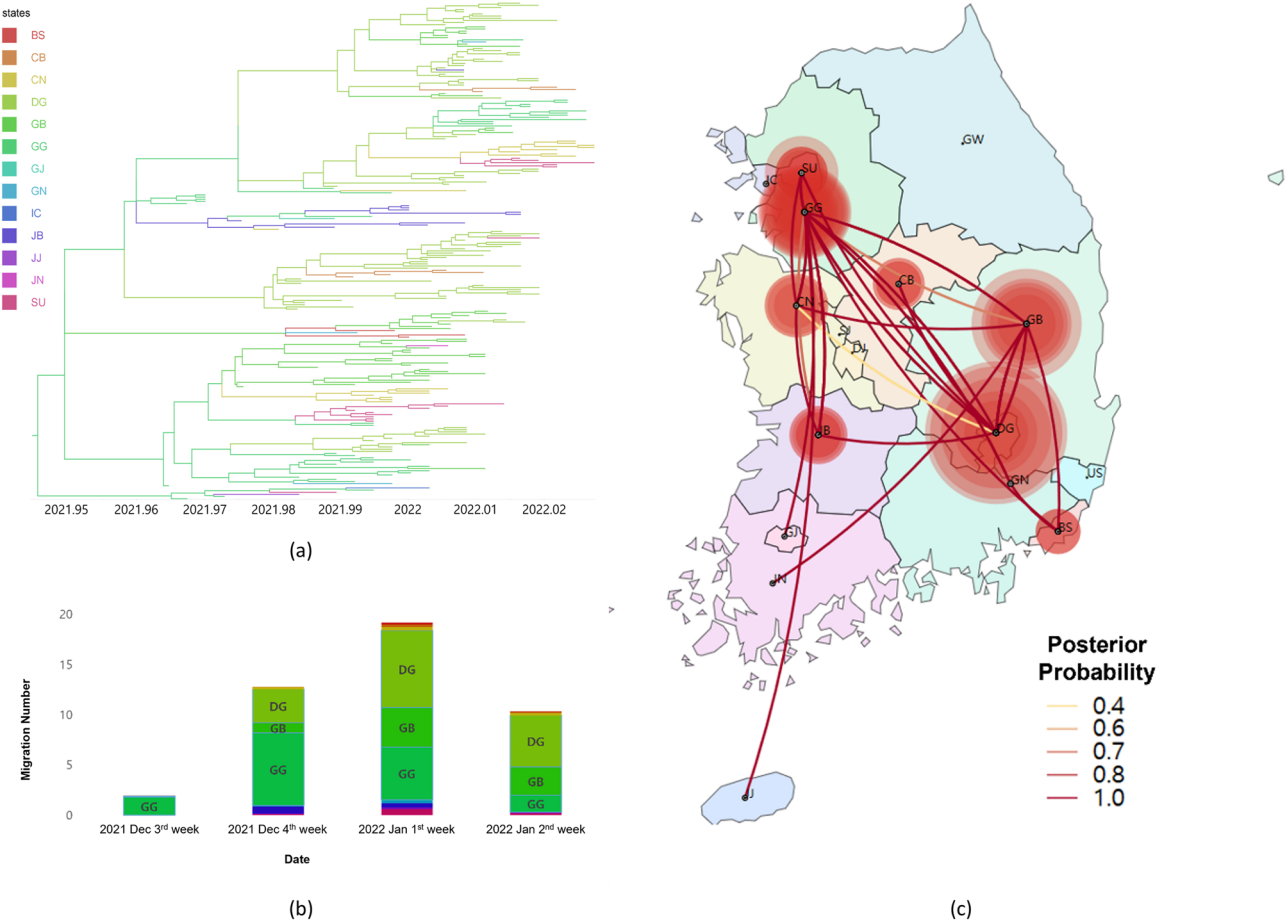
Phylogeographic result of the Kor-O1 subgroup. (**a**) Time-scaled maximum clade credibility tree of the Kor-O1 subgroup. Branches are colored according to their location in South Korea, and the branch thickness indicates the posterior probabilities of the inferred ancestral location. The phylogenetic tree with the virus name is available in Fig. S2. (**b**) Mean number of migration events of the Kor-O1 subgroup from major sources in Korea through time, which was estimated and computed by Bayesian phylogeographic analysis. (**c**) Transmissions of the Kor-O1 subgroup in South Korea visualized by SpreaD3. The color of the lines indicates the posterior probability. The diameters of the circles represent the number of branches maintaining a particular location state in each period. The video of the Kor-O1 transmission history is available on Video S1. (*BS* Busan, *CB* Chungcheongbuk-do, *CN* Chungcheongnam-do, *DG* Daegu, *GB* Gyeongsangbuk-do, *GG* Gyeonggi-do, *GJ* Gwangju, *GN* Gyeongsangnam-do, *IC* Incheon, *JB* Jeollabuk-do, *JJ* Jeju-do, *JN* Jeollanam-do, *SU* Seoul).

To estimate the transmission dynamics of the Omicron variant circulating in South Korea, we reconstructed ancestral locations and inferred migration of viruses for three Korean subgroups (Kor-O1, Kor-O2, and Kor-O3). Our results showed that the three subgroups had different geographic transmission tendencies. The Kor-O1 subgroup spread to 13 out of 17 states. On December 17, 2021, the Kor-O1 subgroup was first detected in Gyeonggi-do, and it was the major site of the outbreak until December 24, 2021. On December 31, 2021, it was primarily spread in Daegu, with an increasing rate of transmission from Daegu and Gyeongsangbuk-do to other regions (Fig. 2, Table 2, and Video S1).

**Table 2:** Migration rates, migration number, Bayes factor, and posterior probability of spread of the Kor-O1 subgroup in South Korea.

From	To	Migration rates^1^ (95% HPD)^2^	Migration number^3^ (95% HPD)	Bayes factor	Posterior probability
GG	JB	1.32 (0–2.83)	2.31 (0–5)	131.15	92.07
	JJ	0.28 (0–1.08)	0.90 (0–1)	13.69	54.80
	DG	2.47 (0–4.85)	3.92 (2–7)	618.18	98.21
DG	SU	1.25 (0–2.84)	3.37 (1–7)	58.22	83.76
	CB	0.58 (0–1.86)	1.94 (0–5)	15.20	57.38
	CN	0.93 (0–2.62)	2.61 (0–7)	19.76	63.64
GB	GN	0.66 (0–2.25)	1.13 (0–3)	13.36	54.20
	JN	0.55 (0–1.58)	0.92 (0–1)	48.39	81.08
JB	GJ	0.60 (0–2.04)	0.36 (0–1)	19.42	63.23

Only well-supported viral migrations (posterior probability > 0.5 and Bayes factor > 3) are displayed in this table.

*GG* Gyeonggi-do, *JB* Jeollabuk-do, *JJ* Jeju-do, *DG* Daegu, *SU* Seoul, *CB* Chungcheongbuk-do, *CN* Chungcheongnam-do, *GB* Gyeongsangbuk-do, *GN* Gyeongsangnam-do, *JN* Jeollanam-do, *GJ* Gwangju.

^1^Actual migration rates were calculated as rate × indicator.

^2^95% HPD, 95% height posterior density.

^3^Number of migrations among regions in phylogenetic tree (Markov jumps) were estimated using stochastic mapping techniques implemented in the BEAST package.

**Figure 3:**
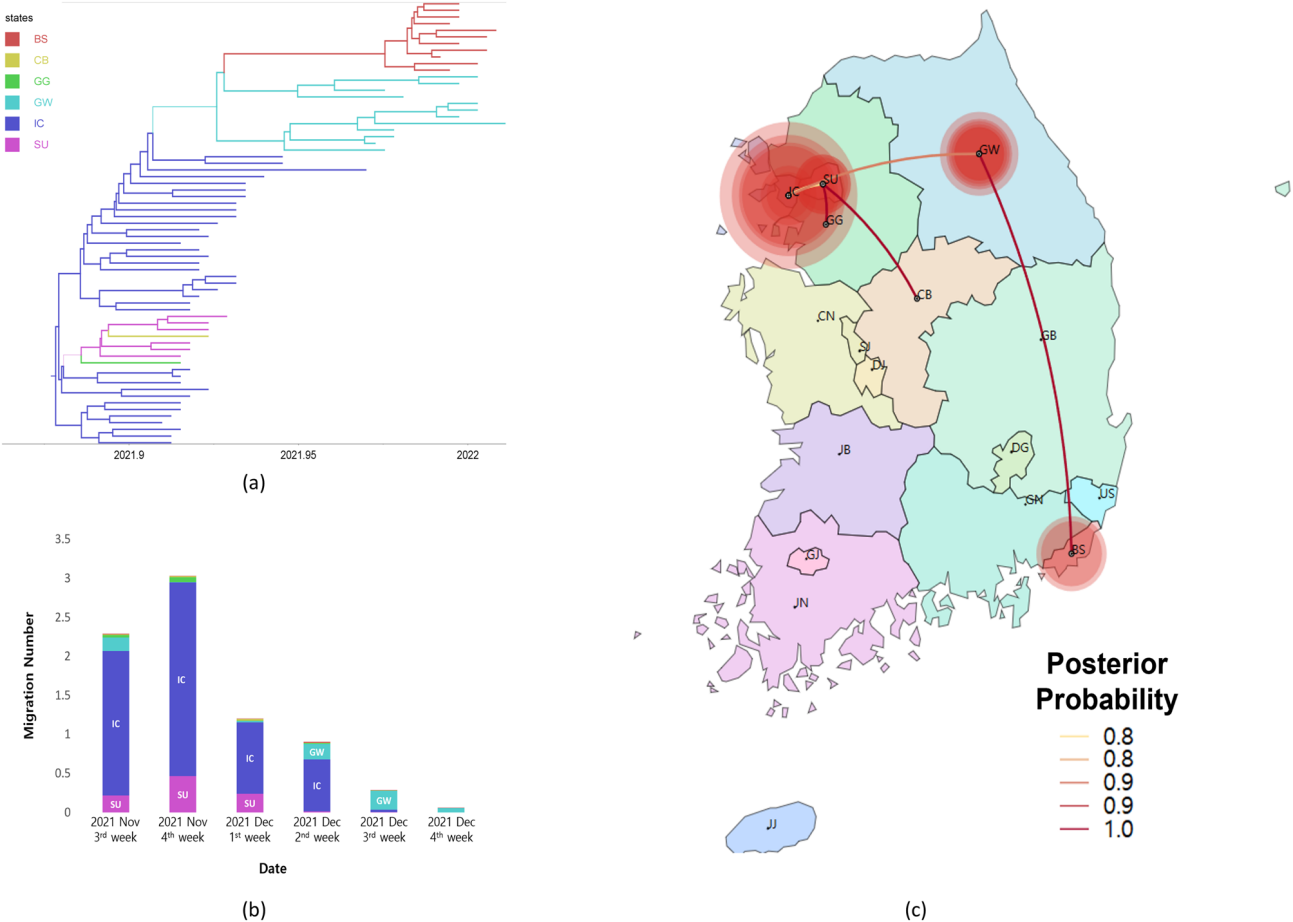
Phylogeographic result of the Kor-O2 subgroup. (**a**) Time-scaled maximum clade credibility tree of the Kor-O2 subgroup. Branches are colored according to their location in South Korea, and the branch thickness indicates the posterior probabilities of the inferred ancestral location. The phylogenetic tree with the virus name is available in Fig. S3. (**b**) Mean number of migration events of the Kor-O2 subgroup from major sources in Korea through time, which was estimated and computed by Bayesian phylogeographic analysis. (**c**) Transmissions of the Kor-O2 subgroup in South Korea visualized by SpreaD3. The color of the lines indicates the posterior probability. The diameters of the circles represent the number of branches maintaining a particular location state in each period. The video of the Kor-O2 transmission history is available on video S2 (*BS* Busan, *CB* Chungcheongbuk-do, *GG* Gyeonggi-do, *GW* Gangwon-do, *IC* Incheon, *SU* Seoul).

In the case of the Kor-O2 subgroup, the virus was primarily detected in three states: Incheon, Gangwon-do, and Busan. Most transmissions started from Incheon to Gangwon-do before mid-December, located in the northern region of South Korea. However, after mid-December, transmission mainly proceeded in Gangwon-do, and nearly all transmissions were directed to Busan, the southern region of South Korea (Fig. 3, Table 3, Video S2).

**Table 3:** Migration rates, migration number, Bayes factor, and posterior probability of spread of the Kor-O2 subgroup in South Korea.

From	To	Migration rates^1^ (95% HPD)^2^	Migration number^3^ (95% HPD)	Bayes factor	Posterior probability (%)
IC	SU	1.27 (0–3.34)	1.63 (0–5)	33.79	88.78
	CB	0.64 (0–2.29)	0.62 (0–1)	6.77	61.34
	GG	0.79 (0–2.42)	0.78 (0–1)	13.38	75.81
	GW	0.97 (0–2.82)	1.16 (0–3)	19.13	81.75
GW	BS	0.71 (0–2.50)	0.51 (0–1)	8.35	66.17
SU	CB	0.64 (0–2.66)	0.35 (0–1)	4.39	50.72

Only well-supported viral migrations (posterior probability > 0.5 and Bayes factor > 3) are displayed in this table.

*IC* Incheon, *SU* Seoul, *CB* Chungcheongbuk-do, *GG* Gyeonggi-do, *GW* Gangwon-do, *BS* Busan.

^1^Actual migration rates were calculated as rate × indicator.

^2^95% HPD, 95% height posterior density.

^3^Number of migrations among regions in phylogenetic tree (Markov jumps) were estimated using stochastic mapping techniques implemented in the BEAST package.

**Figure 4:**
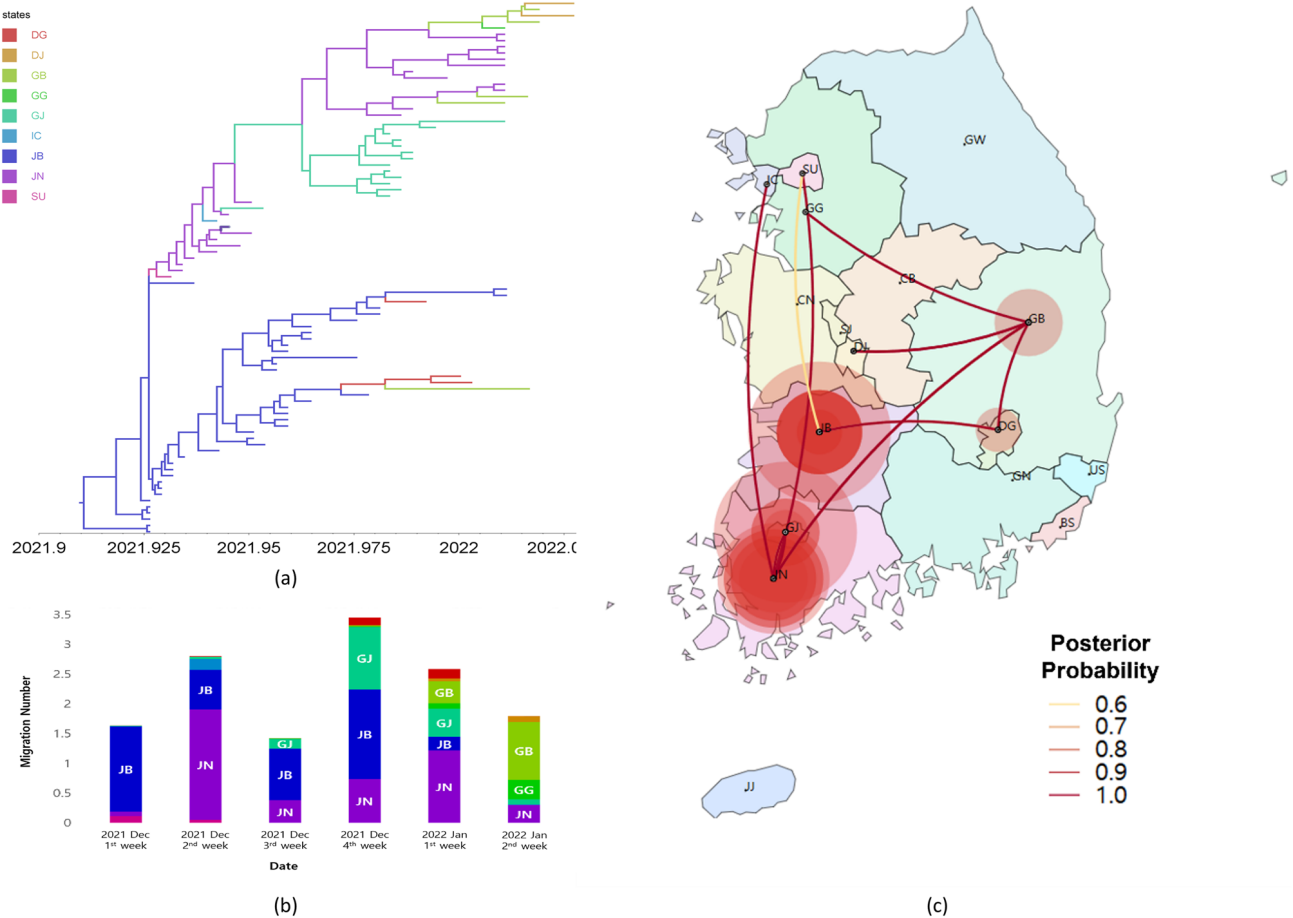
Phylogeographic result of the Kor-O3 subgroup. (**a**) Time-scaled maximum clade credibility tree of the Kor-O3 subgroup. Branches are colored according to their location in South Korea, and the branch thickness indicates the posterior probabilities of the inferred ancestral location. The phylogenetic tree with the virus name is available in Fig. S4. (**b**) Mean number of migration events of the Kor-O3 subgroup from major sources in Korea through time, which was estimated and computed by Bayesian phylogeographic analysis. (**c**) Transmissions of the Kor-O3 subgroup in South Korea visualized by SpreaD3. The color of the lines indicates the posterior probability. The diameters of the circles represent the number of branches maintaining a particular location state in each period. The video of the Kor-O3 transmission history is available on video S3 (*DG* Daegu, *DJ* Daejeon, *GB* Gyeongsangbuk-do, *GG* Gyeonggi-do, *GJ* Gwangju, *IC* Incheon, *JB* Jeollabuk-do, *JN* Jeollanam-do, *SU* Seoul).

Finally, in the case of Kor-O3, we detected that the transmission started in Jeollabuk-do by the end of December. Most transmissions were observed in Jeollanam-do, Jeollabuk-do, and Gwangju, which are in the western region of South Korea. We observed that Kor-O3 in Jeollabuk-do spread mainly to Jeollanam-do, Seoul, and Daegu and that in Jeollanam-do spread mainly to Gyeongsangbuk-do, Gwangju, and Incheon. From the beginning of January 2022, many transmissions were observed from Gyeongsangbuk-do to Gyeonggi-do and Daejeon (Fig. 4, Table 4, Video S3).

**Table 4:** Migration rates, migration number, Bayes factor, and posterior probability of spread of the Kor-O3 subgroup in South Korea.

From	To	Migration rates^1^ (95% HPD)^2^	Migration number^3^ (95% HPD)	Bayes factor	Posterior probability
JB	JN	0.95 (0–2.64)	1.48 (0–4)	32.23	81.57
	SU	0.63 (0–1.91)	0.81 (0–1)	24.34	76.97
	DG	1.16(0–2.67)	1.99 (1–2)	249.76	97.17
JN	GB	1.12 (0–3.27)	1.62 (0–4)	16.17	68.95
	GJ	0.99 (0–2.80)	1.38 (0–4)	29.71	80.31
	IC	0.88 (0–2.26)	0.97 (0–1)	113.13	93.95
GB	GG	0.69 (0–2.54)	0.51 (0–1)	9.56	56.77
	DJ	0.93 (0–2.98)	0.78 (0–2)	15.81	68.46

Only well-supported viral migrations (posterior probability > 0.5 and Bayes factor > 3) are displayed in this table.

*JB* Jeollabuk-do, *JN* Jeollanam-do, *SU* Seoul, *DG* Daegu, *GB* Gyeongsangbuk-do, *GJ* Gwangju, *IC* Incheon, *GG* Gyeonggi-do, *DJ* Daejeon.

^1^Actual migration rates were calculated as rate × indicator.

^2^95% HPD, 95% height posterior density.

^3^Number of migrations among regions in phylogenetic tree (Markov jumps) were estimated using stochastic mapping techniques implemented in the BEAST package.

**Figure 5:**
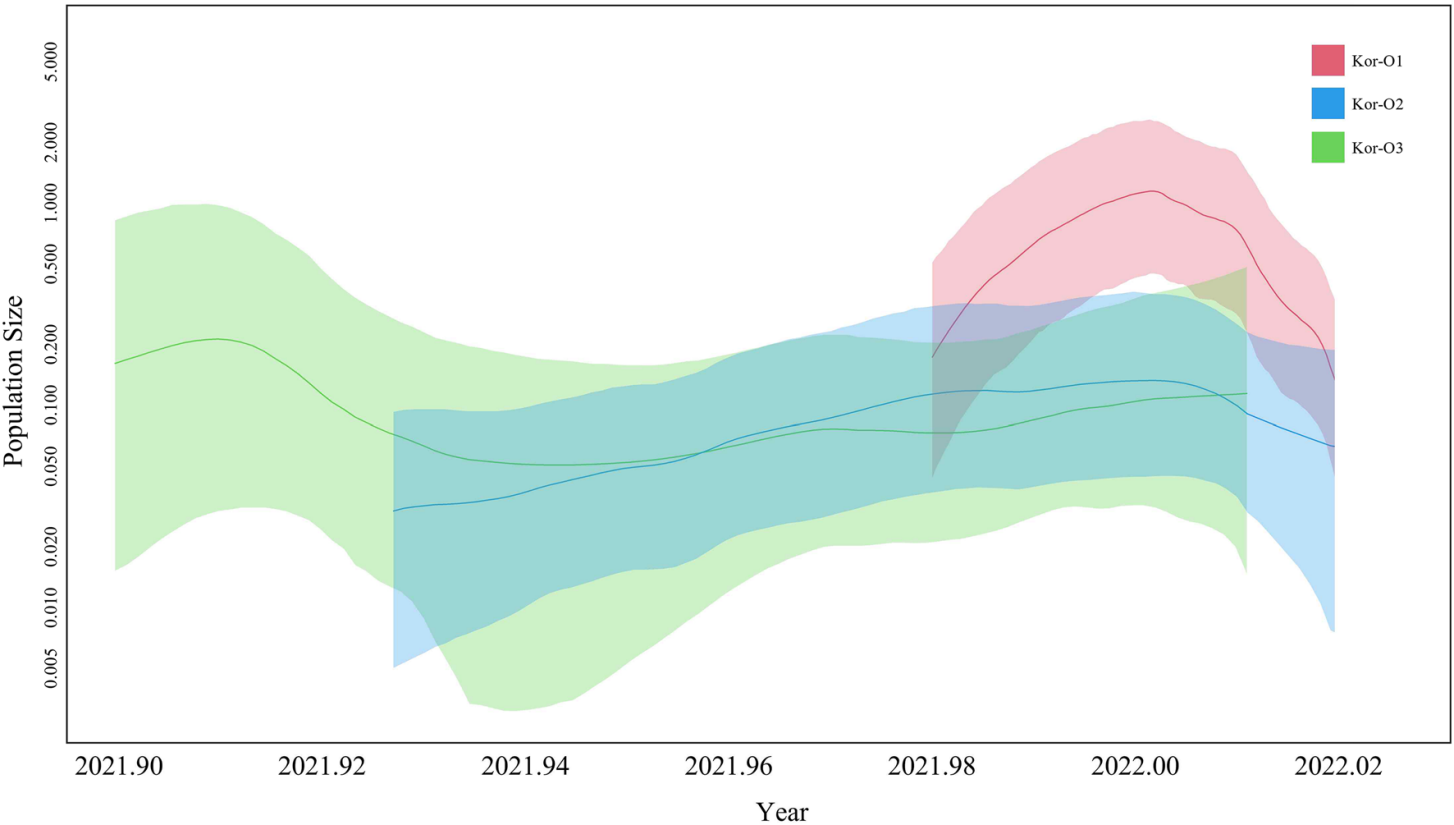
Viral population size change of each Omicron subgroup detected in South Korea. A line indicates the mean value of the viral population size, and 95% HPD is indicated by the solid area.

Population size dynamic analysis showed that the Kor-O2 subgroup was discovered first, followed by Kor-O3 and Kor-O1. Whereas the population size of the Kor-O2 subgroup was constant, the population size of the Kor-O1 and Kor-O3 subgroups continuously increased. Furthermore, the Kor-O2 subgroup was not detected after the last case detected on January 5, 2022 (Fig. 5).

## Discussion

The mutations of the Omicron variant were concentrated in the spike protein, including four deletions and one insertion (Δ69-70, Δ143-145, Δ211-212, ins214EPE)^18,19^. Mutations in the spike protein can enhance the ability of the variant to evade current vaccines^16,20^. In addition, mutations in the receptor binding domain could enhance receptor binding ability, which could affect virus transmissibility^21,22^. The Omicron variant replaced the previous dominant lineage, Delta, and has become the dominant variant worldwide.

Omicron is classified into several sub-lineages (BA.1–5) based on its mutations in PANGOLIN (https://pangolin.cog-uk.io/)^23^. In addition, several recombinant lineages between Delta and Omicron or between BA.1 and BA.2 have been continuously reported^24,25^. The recombinant lineages were not detected in South Korea in this study because we analyzed data collected in the very early stages of the Omicron outbreak. Although BA.2 sequences were also detected, most cases in the early stages of the Omicron outbreak in South Korea belong to BA.1. Therefore, this study focused on BA.1 and its sub-lineage BA.1.1.

South Korea has implemented a 10-day quarantine policy for all inbound travelers since December 3, 2021, to prevent Omicron spread and designated 11 African countries as “Enhanced Quarantine Required Countries” (http://ncov.mohw.go.kr/duBoardList.do#). Despite national border control, our results showed that at least 128 distinct phylogenetic subgroups belonging to BA.1 were introduced in South Korea. Of these, 113 subgroups were introduced into South Korea after implementing border control. The detection of the Kor-O1 monophyletic subgroup from foreign countries near the same time indicated the possibility of the multiple spread of viruses from South Korea to other nations or vice versa. The results indicate that multiple cross-country transmissions of Omicron VOC occurred, despite active border control.

Our study highlights the three Omicron subgroups that co-circulated in South Korea in different regions. These semi-separated circulations might have contributed to the continuous outbreak of each subgroup. As shown in the spread of the Alpha, Delta, and Omicron VOCs, a higher transmissible variant could replace the prevailing strain^26^. Here, the KOR-O2 subgroup was not detected at the end of this study. However, the co-circulation of multiple variants should be carefully monitored. The co-circulation of different variants could be an obstacle to disease control using vaccines and therapeutics. More extended monitoring and continued genomic epidemiology will be required to identify circulating variants.

The Kor-O1 subgroup caused the largest outbreak and spread to most of the regions in South Korea. While the spread of Kor-O2 and Kor-O3 started in Incheon and Jeollabuk-do, respectively, the Kor-O1 subgroup started in Gyeonggi-do. Seoul and Gyeonggi-do are the major metropolitan areas in South Korea and have the largest population and movement of population. Outbreaks in metropolitan regions may contribute to the more frequent and rapid spread of Kor-O1 than that of other subgroups. During our investigation period, the vaccination rate in South Korea was approximately 80%; nevertheless, the Omicron variant rapidly spread and caused a large outbreak. Vaccination could not completely prevent SARS-CoV-2 infection, but the effect of vaccination on the severity and transmission of the disease should be carefully evaluated by further studies.

The ancestral reconstruction and phylogeographic analysis used in this study can be affected by sampling bias^27^. We selected viruses for sequencing based on field epidemiological investigation results to minimize the impact of sampling bias. In addition, we did not conduct down-sampling and used all available sequences to avoid other sampling biases. However, the potential role of unsampled cases and less sampled populations cannot be entirely excluded. Owing to the large outbreak after the introduction of Omicron, sequence analysis was conducted for only approximately 3% of confirmed cases. The possible existence of undetected sub-clinical infections also could not be analyzed. Such sampling biases and low sequencing coverage can affect the results of the phylogeographic analysis. However, this study presents the most probable transmission dynamics of the Omicron variant in South Korea by reconstructing ancestors in phylogenetic analysis, using all of the available sequence data.

As the number of Omicron cases in South Korea increases, it is essential to estimate the spread of variants using molecular epidemiology. However, the early detection of novel variants among the numerous cases can be limited, owing to the restriction of sequencing coverage. We estimate the time of introduction of the virus based on a time-scaled phylogenetic tree, and the results show that South Korea’s current sequence surveillance system could detect new viral introductions within 9 to 12 days.

Omicron variant sub-lineages continue to be detected worldwide, and viruses are evolving into various sub-lineages. As SARS-CoV-2 is becoming endemic in most countries, the Korean government took steps to relax its quarantine policy and reduce the level of social distancing for a gradual return to normal life. Although the number of confirmed cases is gradually decreasing, novel variants of SARS-CoV-2 continuously threaten public health. Because multiple introductions and co-circulation of variants were detected in this study, the relaxed quarantine policy will require improvements in the variant monitoring system and control strategies.

## Methods

### Virus detection and whole-genome sequencing

Nasopharyngeal and oropharyngeal swabs and sputum samples were collected from symptomatic patients to detect SARS-CoV-2 by real-time reverse transcriptase (RT)-PCR. According to the manufacturer's protocol, RNA was extracted from 140 μL of the sample using a Qiagen viral RNA mini kit (Qiagen, Hilden, Germany). Real-time RT-PCR was performed on the extracted RNA and cycle threshold^28^.

The samples were chosen for sequencing from the outbreak data based on epidemiological links. In addition, we selected samples from sporadic cases and a few random representative samples from epidemiologically linked large outbreaks. Whole-genome sequences of selected viruses were analyzed using the ARTIC protocol (https://artic.network/ncov-2019). Libraries were prepared using the Nextera DNA Flex Library Prep Kit (Illumina, USA), and sequencing was performed by the MiSeq instrument using the MiSeq Reagent Kit V2 (Illumina, USA).

After lineage assignment in PANGOLIN (https://pangolin.cog-uk.io/)^23^, we obtained 582 complete genome sequences of Omicron VOC from November 24, 2021, to January 11, 2022, including 550 Korean resident cases and 32 inbound traveler cases (Table S1).

All procedures performed in studies involving human participants were in accordance with the ethical standards of the institution and/or national research committee and with the 1964 Helsinki Declaration and its later amendments or comparable ethical standards. The study was approved by the Institutional Review Board of the Korea Disease Control and Prevention Agency (approval number: 2020-03-01-P-A) and was designated a service to public health during the pandemic. Therefore, the Institutional Review Board waived the requirement for written informed consent, as outlined in the Title Laboratory Respondence to COVID-19.

### Identify phylogenetic subgroups

BA.1 and BA.1.1 sub-lineages of Omicron full-genome sequences, identified as of January 31, 2022, in other countries, with > 29,000 nt and < 1% undefined bases, were downloaded from the GISAID EpiCoV™ database (https://www.gisaid.org/) for reference sequences (n = 7053). For efficient computation and analysis, we selected 3330 sequences (1543 from Europe, 951 from North America, 543 from Asia, 212 from Oceania, 54 from Africa, and 27 from South America) based on the nucleotide sequence identified at the 99.97% level using the program CD-HIT^29^. We used Nextclade v1.13.2 (https://clades.nextstrain.org/) for quality control (QC) of 582 sequences isolated in South Korea. Nextclade uses an algorithm that considers missing data, mixed sites, private mutations, mutation clusters, stop codons, and frameshifts in calculating a score. A total of 32 sequences that exceeded the QC score of 100 were excluded, resulting in 550 sequences remaining.

Using Geneious Prime software (https://www.geneious.com/), each sequence was aligned to the reference sequence Wuhan-Hu-1 (GenBank ID MN908947). Aligned sequences were trimmed to equal lengths (29,409 bp) from the start codon of ORF1ab to the stop codon of ORF10. Frame shifting insertions in < 1% of sequences were considered erroneous sequencing results and were deleted through mask alignment in the Geneious software. The approximately maximum-likelihood phylogenetic tree was constructed using FastTree in Galaxy Europe (http://usegalaxy.eu/) under the general time reversible (GTR) model for the effective computation of big genomic data. The phylogenetic tree was visualized and annotated using iTOL (https://itol.embl.de/). In the FastTree result, we divided Korean sequences into three subgroups with the monophyletic cluster sharing a common ancestral node with other Korean sequences showing a local support value of > 0.7. This resulted in a Kor-O1 subgroup with 273 sequences, a Kor-O2 subgroup with 68 sequences, and a Kor-O3 subgroup with 86 sequences.

To investigate the mutations of each subgroup of Korean sequences, we compared the Korean sequences with the first isolated Omicron, hCoV-19/South Africa/NICD-N20868/2021, collected on November 11, 2021, as a reference sequence.

### Phylogeography and population size dynamics of each subgroup

For each Korean sub-lineage, we reconstructed the maximum-likelihood phylogenetic tree using Geneious Prime software, and the temporal signal of the dataset was analyzed using the TempEst program (https://beast.community/tempest)^30^. Outlier sequences in the root-to-tip regression were excluded. In addition, we deleted 2 sequences of inbound travelers and 35 reference foreign sequences from the data to focus on the geographical relationships in virus transmission in South Korea. The final data set of the Kor-O1 subgroup with 231 sequences, the Kor-O2 subgroup with 67 sequences, and the Kor-O3 subgroup with 86 sequences were used for time-scaled phylogeographic analysis.

To estimate the transmission and introduction of Omicron in South Korea, we constructed a time-scaled phylogenetic tree for three subgroups (Kor-O1–Kor-O3) using BEAST v.1.10.4^31^. The sequences of each subgroup were coded as 17 states and other geographic locations by the administrative district in South Korea. Posterior phylogenetic tree distribution was estimated using Bayesian phylogenetic inference. The general time reversible model, along with a gamma-distributed rates (GTR + γ) nucleotide substitution model was selected. To estimate the viral population size change, the unweighted pair group method with an arithmetic starting tree model and Bayesian Skygrid coalescent tree prior was used with an uncorrelated relaxed clock model. For each subgroup, Markov-chain Monte Carlo was run in parallel for four chains, each with 150 million steps. The parameters and trees were sampled every 10,000 steps, yielding a total of 60,000 parameter states and posterior trees. The parameters were analyzed using TRACER v1.5 (https://beast.community/tracer)^32^ and burned in 10%–20% of each result. All parameters had an effective sample size of > 200 (Table S2). The log and tree files of each subgroup’s resulting data were combined with LogCombiner v1.10.4 (https://beast.community/logcombiner). The combined log file was analyzed using TRACER 1.7.2 to reconstruct the effective population size over time that was estimated by the Gaussian Markov random field skyride model. A time-scaled maximum clade credibility tree was generated using TreeAnnotator v1.10.4 (https://beast.community/treeannotator) in BEAST and visualized using FigTree 1.4.3 (http://tree.bio.ed.ac.uk/software/figtree/), and the migration routes were visualized using SpreaD3 v0.9.6 (https://beast.community/spread3)^33^.

The rate and number of transitions among the regions (Markov jumps) were estimated using stochastic mapping techniques implemented in the BEAST package^34^. Posterior trees were analyzed using the PACT program (http://www.trevorbedford.com/pact) to compute the number of transition events between the regions through time. The posterior trees were broken into multiple temporal sections (one week per section). The migrations of the location state at the tree nodes were counted at each time window for each posterior tree.

## Supplementary Information

Supplementary Information.

## Data Availability

The sequences were shared through the GISAID EpiCoV (https://www.gisaid.org/) database. The list of accession numbers of South Korean SARS-CoV-2 sequences used in this study is available in Table S1.
