# Supplementary material for: Genomic epidemiology of SARS- CoV-2 Omicron variants in the Republic of Korea
Source: Sci Rep. 2022 Dec 27;12:22414. doi: 10.1038/s41598-022-26803-w (PMC9793390; doi:10.1038/s41598-022-26803-w)
Supplement: Supplementary file 1 — Supplementary Information. [file 41598_2022_26803_MOESM1_ESM.zip › Supplementary Information and figures.docx]

**Supplementary Information**

**Table S1.** List of South Korean SARS-CoV-2 strains used in this study and their metadata.

**Table S2.** Effective sample size of Bayesian phylogenetic analysis using BEAST 1.10.4v program.

**Video S1.** Animation of Figure 2, panel C. Spatiotemporal reconstruction of the spread of the Kor-O1 subgroup in South Korea.

**Video S2.** Animation of Figure 3, panel C. Spatiotemporal reconstruction of the spread of the Kor-O1 subgroup in South Korea.

**Video S3.** Animation of Figure 4, panel C. Spatiotemporal reconstruction of the spread of the Kor-O1 subgroup in South Korea.

**Supplementary Figures**

**
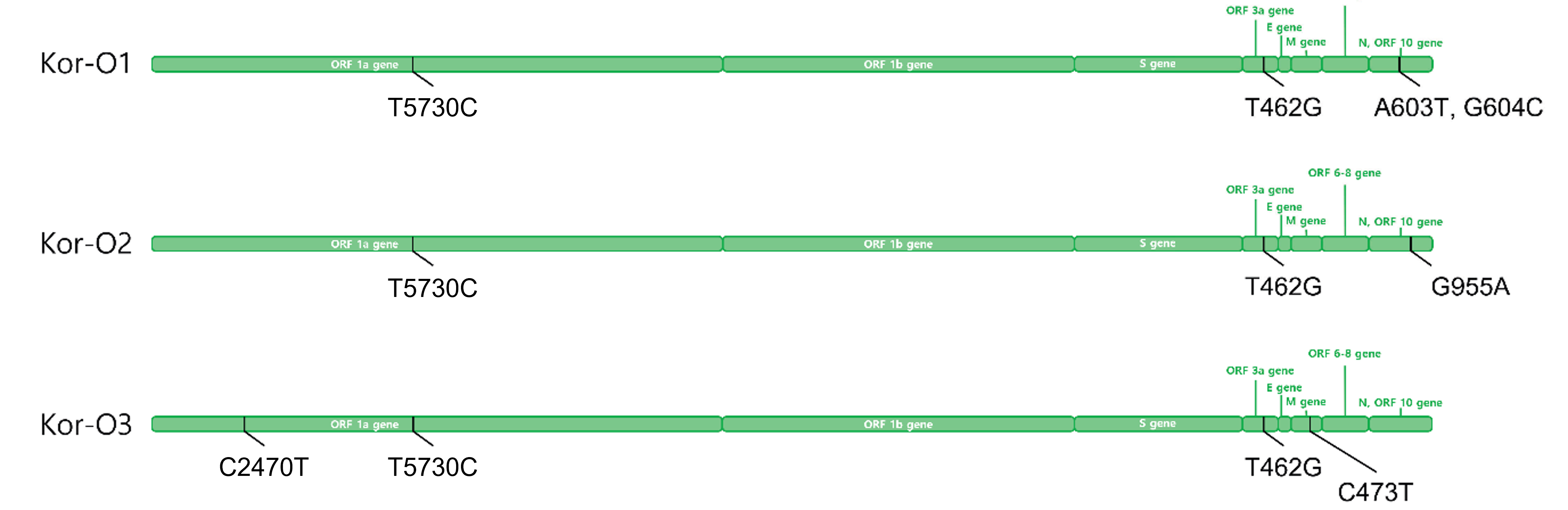
**

**Figure S1.** Mutation of Kor-O1, Kor-O2, and Kor-O3 subgroups circulating in South Korea. First Omicron strain, hCoV-19/South Africa/NICD-N20868/2021, collected on November 11, 2021, was set as a reference sequence and the mutations from the reference strain were annotated.





**Figure S2.** Time-scaled maximum clade credibility tree with virus name of the Kor-O1 subgroup.





**Figure S3.** Time-scaled maximum clade credibility tree with virus name of the Kor-O2 subgroup.


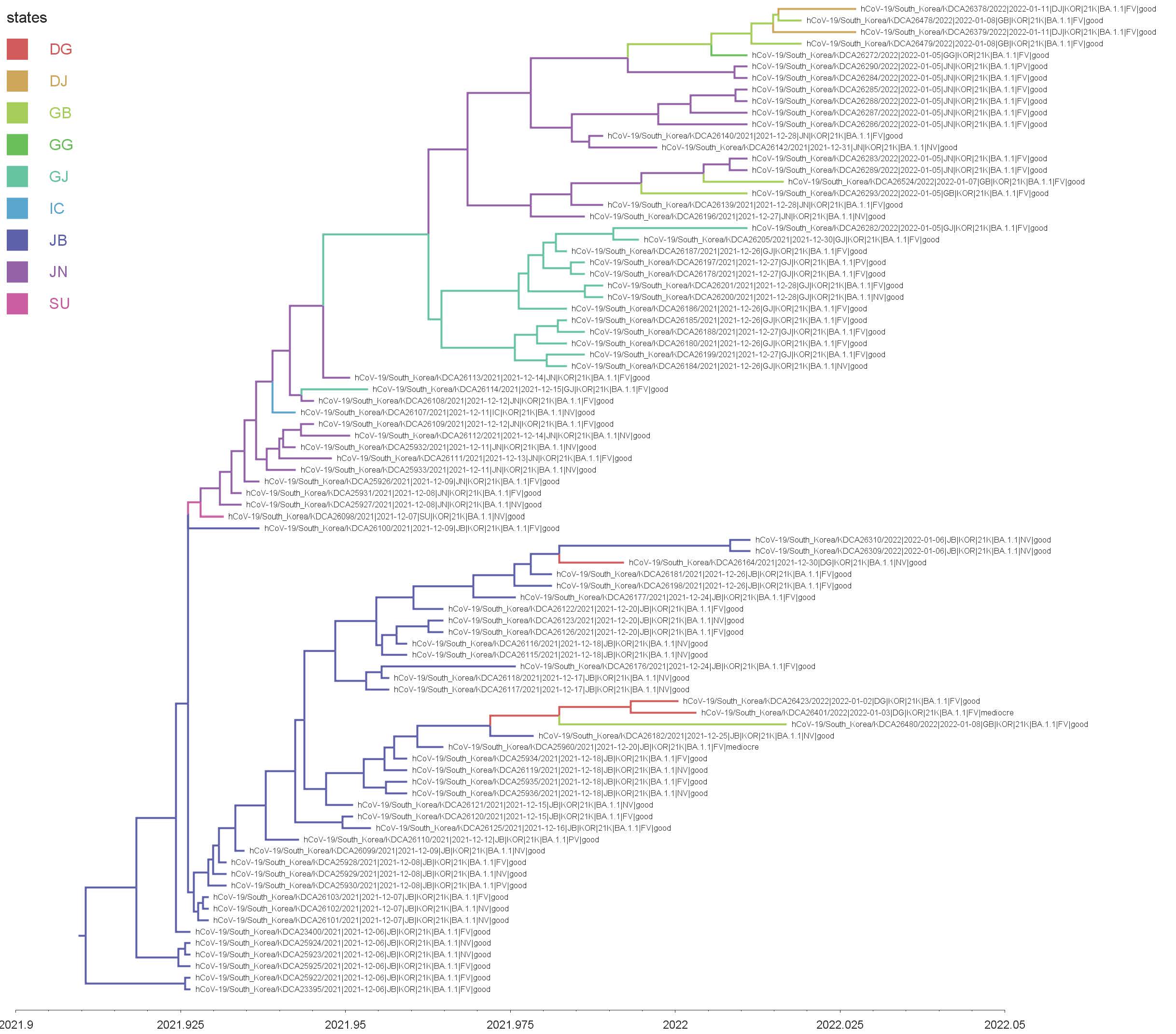


**Figure S4.** Time-scaled maximum clade credibility tree with virus name of the Kor-O3 subgroup.
