# Supplementary material for: Genomic epidemiology of SARS- CoV-2 Omicron variants in the Republic of Korea
Source: Sci Rep. 2022 Dec 27;12:22414. doi: 10.1038/s41598-022-26803-w (PMC9793390; doi:10.1038/s41598-022-26803-w)
Supplement: Supplementary file 1 — Supplementary Information. [file 41598_2022_26803_MOESM1_ESM.zip › Supplementary Table S2.docx]

**Table S2.** Effective sample size of Bayesian phylogenetic analysis using BEAST 1.10.4v program

| **Data set** | Kor-O1 | | Kor-O2 | | Kor-O3 | |
| --- | --- | --- | --- | --- | --- | --- |
| **Total states** | 540040000 | | 405030000 | | 540040000 | |
| **Statistic** | Mean | ESS | Mean | ESS | Mean | ESS |
| joint | -44042.136 | 1133.283 | -40706.107 | 292.727 | -41073.612 | 4753.077 |
| prior | -2884.138 | 1045.826 | -548.598 | 278.795 | -802.135 | 3350.625 |
| likelihood | -41157.998 | 1254.725 | -40157.508 | 4808.518 | -40271.477 | 3458.859 |
| treeModel rootHeight | 7.76E-02 | 5868.837 | 0.133 | 1422.678 | 0.12 | 11167.21 |
| age(root) | 2021.95 | 5868.837 | 2021.878 | 1422.678 | 2021.908 | 11167.21 |
| treeLength | 2.37 | 886.217 | 0.924 | 679.92 | 1.098 | 2767.675 |
| skygrid. precision | 0.548 | 38616.928 | 57.08 | 6250.69 | 7.215 | 19069.477 |
| gtr.rates. rateAC | 0.452 | 11182.732 | 0.217 | 30004.13 | 0.355 | 51218.773 |
| gtr.rates. rateAG | 0.995 | 2265.538 | 2.406 | 15674.643 | 1.675 | 32602.631 |
| gtr.rates. rateAT | 3.91E-02 | 25490.439 | 0.414 | 32284.655 | 0.11 | 35348.874 |
| gtr.rates. rateCG | 0.194 | 49915.323 | 0.294 | 31178.098 | 0.246 | 45483.689 |
| gtr.rates. rateCT | 3.857 | 3553.718 | 1.889 | 22837.171 | 2.826 | 40766.555 |
| gtr.rates. rateGT | 0.463 | 9946.545 | 0.78 | 33642.621 | 0.788 | 50072.042 |
| frequencies1 | 0.299 | 53909.927 | 0.299 | 36453 | 0.299 | 54004 |
| frequencies2 | 0.182 | 53766.806 | 0.183 | 36386.332 | 0.183 | 52726.71 |
| frequencies3 | 0.196 | 52616.553 | 0.196 | 36453 | 0.196 | 54004 |
| frequencies4 | 0.323 | 53196.068 | 0.322 | 36453 | 0.322 | 54004 |
| alpha | 6.05E-02 | 53381.611 | 0.182 | 31625.904 | 0.154 | 47567.73 |
| ucld.mean | 1.49E-03 | 1959.266 | 1.30E-03 | 5154.2 | 1.12E-03 | 10953.757 |
| ucld.stdev | 2.79E-03 | 5329.912 | 7.56E-03 | 18439.653 | 1.60E-03 | 24256.906 |
| meanRate | 1.34E-03 | 1479.389 | 9.61E-04 | 1412.734 | 9.87E-04 | 5147.753 |
| Coefficient Of Variation | 1.473 | 5503.777 | 1.385 | 10093.83 | 0.804 | 11560.547 |
| covariance | 7.46E-03 | 30650.178 | -7.77E-03 | 35312.504 | -1.85E-03 | 52348.73 |
| Tree Likelihood | -227.082 | 967.116 | -30.453 | 2136.049 | -60.939 | 5241.494 |
